# Smallest detectable differences in clinical variables related to temporomandibular joint arthritis in juvenile idiopathic arthritis

**DOI:** 10.1186/1546-0096-9-S1-P127

**Published:** 2011-09-14

**Authors:** Peter Stoustrup, Carlaberta Verna, Kasper D Kristensen, Annelise Küseler, Troels Herlin, Thomas K Pedersen

**Affiliations:** 1 Department of Orthodontics, Faculty of Health Sciences, University of Aarhus, Denmark; 2 Department of Pediatrics, Aarhus University Hospital, Aarhus N, Denmark

## Background

Temporomandibular joint (TMJ) arthritis in juvenile patients may lead to craniofacial growth deviations as well as interfere with optimal joint function and mouth opening pattern. Clinical assessment of maximal mouth opening capacity, laterotrusion and protrusion is widely used in the diagnosis of TMJ arthritis as well as in the evaluation of a therapeutic intervention in spite of missing knowledge about the reproducibility of these measurements.

## Aim

To calculate the smallest detectable differences in terms of maximal mouth opening capacity, laterotrusion and protrusion. The smallest detectable difference is the minimal amount of change that can be identified clinically between two consecutive observations.

## Methods

42 patients with juvenile idiopathic arthritis (JIA) were included (mean age 12.8, females 73.8%, previous/current TMJ arthritis 57.1%). During their clinical orthodontic examinations repeated measurements of maximal mouth opening capacity, laterotrusion and protrusion were conducted by two experienced observers with a calibrated ruler. The measurements were repeated three times by each observer. Intra- and inter-observer variations were calculated. Variance components were identified by analyses-of-variances and used for the calculation of the smallest detectable differences for each of the variables examined.

## Results

The smallest detectable differences found were: maximal mouth opening capacity 7 mm, laterotrusion 3 mm and protrusion 4 mm. These values were reduced if repeated measurements were conducted. No relationship between the age of the patient and the measurement reproducibility was observed.

## Conclusion

This is the first study to establish guidelines for the detection of genuine changes between separate clinical TMJ examinations in JIA patients.

